# Does Acute General Dropsy Ever Occur as an Idiopathic Disease?

**Published:** 1878-05

**Authors:** J. A. Goldsberry

**Affiliations:** Annapolis, Ind.


### DOES ACUTE GENERAL DROPSY EVER OCCUR AS AN IDIOPATHIC DISEASE?

By J. A. Goldsberry, M. D., Annapolis, Ind.

Dr. Flint says that "dropsy is always dependent on some antecedent morbid condition; that it is never a primary affection; that it is, in fact, not a disease, *per se*, but only a symptom of disease."

Dr. Hughes Bennett says "that serous effusion, or dropsy, is always indicative of mechanical obstruction to the return of the blood from the capillaries through the veins." So far as I have any knowledge, this is the theory taught in all our text books on disease, and the belief of the profession generally. Of course it is no purpose of mine, in what I may say, to call in question the fact, long since established by competent observers in the field of pathology, that acute dropsy, as a rule, is wholly symptomatic, and that it occurs as a result of some antecedent diseased condition. But the questions I would ask in all candor, and which I would be pleased to have answered to my entire satisfaction, are: Does it *always* depend upon mechanical obstruction? Does acute dropsy, characterized by general anasarca, always depend on that condition of the kidneys known as desquamative nephritis? Is it not possible, or indeed quite probable, that, now and then, the disorder may be, in every sense of the word, a disease; that it may exist as a primary or idiopathic affection, and that many of the cases of acute albuminuria, so-called, are not albuminuria, do not originate in renal disease, but have their origin in, and are dependent upon, some general constitutional irritation?

It is, perhaps, not very difficult for us to understand why serous effusion should be a prominent symptom in true acute Bright's disease, nor why it should exist in the chronic form of that affection, when the functions of the kidneys have become seriously impaired by destructive structural changes. Nor is it a fact difficult of solution that dropsy *may* follow, sooner or later, organic disease of the lungs, heart or liver. But when we come to treat of dropsy as an idiopathic disease, we confess that the serous transudation is not so easily accounted for. Is it certain that disease may not sometimes be mistaken for symptoms? and does it necessarily follow, because the pathology of certain morbid phenomena cannot be clearly made out, that therefore disease is not present? Certainly not. As already remarked, the profession may be said to be almost a unit in the belief that acute general anasarca is of renal origin, that it is absolutely the offspring of diseased kidneys.

But what is the nature of this disease? What are some of its pathological characters? Johnson tells us that it is an "acute inflammation of the membrane lining the convoluted tubes, and as a result, a prominent feature is the desquamation of the renal epithelium," hence the appropriate name, *Acute desquamative nephritis.* He regards the secreting cells as the primary seat of the local morbid disorder. "The obstruction of the tubes and the loss of the secreting cells, lead to the transudation of serum, causing the albuminuria, and to the deficient elimination of urea, causing, in some instances, uræmic poisoning. The diminished density of the serum from the loss of albumen, together with the embarrassment of the capillary circulation from the retention of urinary principles, occasion the dropsy."

It is not necessary that I should refer to the anatomical changes presented by the kidneys, nor that I should detail the chemical history of the disease. I speak specially of one prominent feature or symptom, which, so far as I know, is invariably present, a symptom, by which, in every instance, the affection is characterized—I allude to the presence of albumen in the urine. In speaking of the disease in connection with albuminous urine, Flint observes, "The urine is found to contain albumen, usually in considerable, and frequently in great abundance."

Geo. B. Wood says, "The urine is highly albuminous; in some cases so abundant in albumen, that it is converted by the coagulation of that principle, into a uniform jelly; and the deposit, upon standing twenty-four hours after coagulation, seldom occupies less than one-third of the bulk of the fluid."

Roberts goes farther still, and declares that in some cases albumen may be present in such enormous quantities, that "the urine becomes quite solid on boiling."

Bearing in mind then, that acute dropsy, depending upon desquamative nephritis, is albuminuria; that in no case can the disease occur, whether it come up as a result of diseased kidneys, or as a sequel to some other morbid condition, without the presence of albumen in the urine, how are we to account for the cases of acute general anasarca or dropsy, so frequently met with, which are not characterized by the presence of this principle in the urine, and cannot therefore be of renal origin? It is a fact well known to every observant physician, who has had more or less experience in the treatment of scarlatina, that one of its most frequent sequels is general dropsy, and if we would accept the teachings of our books, we must believe that the serous effusion in these cases, is caused by vascular mechanical impediment, and that that impediment depends, primarily upon albuminous nephritis; in other words, that in scarlatinous dropsy we have precisely the same pathological condition of the kidneys which obtains in acute albuminuria. My own experience with the dropsy of scarlet fever has been limited, and as some of the main points in the clinical history of the cases, of which, from first to last, I have had control, were not regularly noted, I cannot speak with positiveness as to the character of the urine. But in this connection I am fortunate in being able to call attention to authority which, to say the least, is, I think, entitled to our respect and earnest consideration.

As far back as 1814, Dr. Blackall, an English physician and author, reports in his "Observations on the Nature and Cure of Dropsy," the following cases:

"Case 1. M. T., æt. 12, was just recovering from scarlatina, in which the inflammation of the skin had been severe, and the bowels unusually relaxed. On the evening preceding my visit, the ankles and knees had begun to swell. There was a considerable degree of languor and loss of appetite; a quick and weak pulse; pain of the left side; a loose state of the bowels and swelling of most of the joints, particularly the knee, in the large bursa mucosa, above which there was very evident fluctuation. The urine was rather scanty, pale and without sediment. It was coagulable neither by heat nor nitric acid. True dropsical symptoms succeeded rapidly.

In less than a fortnight she became universally anasarcous, and there was fluctuation of water in the abdomen, with orthopnea and frightful dreams. At length she spent several successive nights in her chair, unable to lie down. It was impossible to entertain any doubt of the presence of hydrothorax. During the whole of this time the urine, examined daily, gave no appearance of coagulum. She finally made a complete and rapid recovery by the use of smart purges of jalap and scammony and Peruvian bark.

"Case 2.—S. S., æt. 45, Devon and Exeter Hospital. Considerable anasarca, with a feeble pulse and loss of appetite; urine pale, rather scanty, not coagulable by heat or nitric acid and depositing no sediment. Six weeks before, she had been attacked by fever and a scarlet eruption, to which her dropsical symptoms had succeeded in a few days. She derived immediate advantage from bitter infusions and alkaline salts and soon recovered."

Referring to these cases of Blackall, Royer says: "I have known many similar ones, but as none have died, I have been uncertain as to the cause or mode of production of the dropsies." The existence of scarlatinal anasarca, independent of diseased kidney, is emphatically asserted by Barthez and Rilliet (see Traité des Maladies des Enfants) who state that they "have seen one fatal case, and established not merely by investigation during life, but also by examining the glands themselves after death, that the kidneys were not affected." Becquerel and Rodier insist that cases of dropsy following scarlet fever without albuminous urine are not rare." In the Dublin Medical Press for 1866, three cases of scarlatinous dropsy without albuminuria, are related by Dr. G. S. Smith. Dr. Stiebel, a distinguished German physician, states that he "has seen more cases of scarlet-fever dropsy without, than with albumen. If the average of observed cases of scarlet-fever dropsy be taken, it will be found that in not more than one-third of the cases is albumen present." Dr. Niemeyer, than whom there could perhaps be no higher authority, says: "The second form of scarlatinous dropsy, not accompanied with albuminuria, is a sequela of scarlet fever, as free from danger as it is inexplicable. It generally develops gradually, may become very extensive and is not limited to the subcutaneous cellular tissue. In some cases of scarlatinous dropsy without albuminuria, recovery takes place in a remarkably short time, as I know from personal experience." According to the testimony of authority already quoted, we may have, very rarely however, cases of albuminous nephritis, following scarlet fever, without dropsy. Simon says (see Simon's Chemistry, American Edition), we have dropsical symptoms with albuminuria, dropsical symptoms without albuminuria, and albuminuria without dropsical symptoms. Referring to dropsy in connection with scarlatina, and the facts above stated, H. C. Wood, in the *Am. Journ. of the Med. Sci.* for 1870, gives it as his opinion that inasmuch as it is established "that either dropsy or albuminous nephritis may exist alone after scarlet fever, but are in the vast majority of cases associated together; that either may precede the other; that they are often simultaneously and rapidly developed after exposure to a sudden malign influence—it follows, he thinks, that neither is the cause of the other, but that they are both the results of a common cause—a common irritation." In the opinion of many, this theory doubtless would be open to criticism, but it seems to me that there is something rational in it and altogether consistent with the principles of pathology. I come now to speak of acute dropsy as a primary affection—as an idiopathic disease, if the term be preferable; and in doing so, I will summon to my aid facts obtained from the clinical experience of others who are much more competent to speak upon the subject under consideration than myself. Dr. Edward J. Seymour, writing as long ago as 1837, (see The Nature and Treatment of Dropsy), pretty accurately describes the most prominent features of the affection. His language is as follows:

"In some cases, after sudden exposure to cold and wet, the whole cellular membrane becomes infiltrated, the swelling is hard and tense, the pulse hard, the urine scanty and incoagulable by heat or acids, the heart beats without symptoms of organic disease though its action be increased, the bowels are costive, the effect likewise follows very rapidly on the cause applied; the patient's health has been good. There is in this state a general feverish excitement. The cure of this form of disease will generally be perfected in about ten days."

The following case was published, with clinical remarks, by Dr. W. R. Basham in the *London Lancet* for 1867. More reliable authority on any point relating to dropsy, it would, perhaps, be difficult to find.

"R. H., æt 26, admitted to hospital January 22. About a fortnight previously, having been exposed to wet and cold, he had chills, pains in limbs and fever and, a morning or two afterwards, suffered from difficult breathing, with swollen, puffy state of the face and of the surface of the body generally. On admission, the aspect of the patient was strictly characteristic of acute albuminuria. The face was puffy; the eyelids were œdematous, as well as the backs of the hands and the arms as far as the elbows; the surface of the chest was slightly anasarcous, but the dropsy was more pronounced from the thighs downwards. The respirations were hurried and the breathing movements short; a deep inspiration could not be taken. The breath-sounds throughout the chest were indistinct, and everywhere obscured by moist, wheezy murmurs. The resonance was equal in the corresponding regions of the two sides. The sounds of the heart were indistinct, and everywhere obscured by moist chest sounds. Pulse, ninety. He complained of a teasing cough, worse at night, and dyspnœa, coming on in paroxysms. The expectoration was bronchial mucus. There was free micturition, but the urine was scanty in quantity, very high colored, of a bright sherry color, and of sp. gr. 1022. Not a trace of albumen could be detected in it. Neither heat and nitric acid, nor nitric acid alone poured gently down the side of the tube so as to form a layer at the bottom, indicated the faintest trace; nor was any cloud produced by nearly pure alcohol. He was treated by purgatives and diuretics, and in nine days the dropsy had disappeared. The urine was carefully examined every day, but no albumen was discovered. A faint pericardial murmur was found in the heart. It was distinctly to and fro, but was unaccompanied by any pulse affection, which was from 70 to 80, or embarrassed breathing after dropsy was relieved. The pericarditis was believed to date back to a previous attack of rheumatism. The man left the hospital perfectly recovered, eighteen days after admission."

The following case treated and reported as one of idiopathic dropsy, by H. C. Wood, may be found in the *Am. Journ. of the Med. Sci.* for 1871 :

" The patient was a lady of about forty years of age, whose family physician I had been for some years. She was free from all organic disease. About two years before, she had had a miscarriage, but, excepting at that time, and whilst she was suffering from some uterine cervicitis produced thereby, she had enjoyed good health ever since I knew her. Oct. 25, 1870, I was called to see her. During the summer she had suffered greatly from the excesssive heat, but had not been sick. For the last week or two her friends had told her that she was looking very full in the face, but she herself had perceived nothing until the previous Thursday, when, on attempting to put her shoes on she found her feet so swollen as to prevent her. By Sunday she was in the same condition as at present. Present condition : Face and legs very highly œdematous ; trunk and arms very decidedly so. Face somewhat pale, but there are no marks of anæmia. Tongue contracted, firm without teeth-marks. Heart-sounds normal; bowels normal; urine, according to her statement, passed in normal amount, limpid, free from sugar or albumen. Ordered, potass. bitart. ℥j. inf. juniperi oj. Take in 24 hours. 28th. Swelling gone from face; nearly so from feet. 30th. Patient well.

The following is a case of my own, whose clinical history, in almost every particular, corresponds with the two cases just cited:

On the morning of Nov. 15th, 1877, J. T., living several miles in the country, brought to my office his child, a boy about four years of age, for examination and such treatment as, in my opinion, he might need. The little fellow was quite bright and lively and seemed to be wholly unconscious of the slightest physical ailment, and had it not been for the very perceptible œdema of the face and hands, which at once attracted my attention, I would have thought any special examination of the case quite unnecessary. The eyelids and face were considerably puffed; the hands were swollen, the arms, however, were not affected. No anasarca of the surface of the body was observable. The feet were swollen and tense, and the legs more or less œdematous; no swelling of the thighs. The prepuce and scrotum were also involved in the effusion to a limited extent. There was slight cough, which seemed to depend entirely on bronchial irritation. Respiratory murmur plainly audible over both lungs; heart-sounds, distinct and normal. Respirations somewhat quickened. Pulse, 105; full and strong; tongue, very slightly furred. Urine, rather scanty, but in other respects apparently normal. Bowels, constipated. The child was fat and muscular, was well and compactly built, and was evidently the possessor of a fine constitution. On making inquiry as to his previous history, the father informed me that all his life long he had enjoyed uninterrupted good health, and not until the present attack of illness, which was noticed for the first time two or three days previous, had the child been sick even a single day. Nov. 17. The father called and reported the case not so well; he was certain that the swelling was rapidly increasing, with an aggravation of the symptoms generally.

Nov. 19th. I was requested to visit the patient; found him much worse. The anasarca had become general, and, in degree was absolutely frightful. The feet, legs and thighs, and hands and arms were enormous in size, and by reason of their great weight, were almost useless.

The increased swelling of the face had almost obliterated the eyes, and the integument covering the body seemed stretched to its utmost capacity. The scrotum had grown to an incredible size and the penis was simply without form—an irregular, shapeless mass, having lost every vestage of its original identity. There was hydrothorax, marked and decided; effusion into the abdominal cavity not clearly made out; heart-sounds scarcely perceptible; respiratory murmur very feeble, with exaggerated bronchial respiration. Breathing rapid; a moist, wheezing harrassing cough with paroxysms of dyspnœa not unlike the paroxysms of asthma. Pulse, 120; temperature, 102°; quite thirsty. Bowels still disposed to constipation. No gastric disturbance of any consequence. Urine still deficient in quantity, very nearly natural in color, and with a specific gravity of 1,023. From time to time during the progress of the case, the urine was very carefully examined with heat and nitric acid, with always the same unvarying result—a complete absence of even the slightest trace of albumen.

The treatment of this little sufferer, from first to last, consisted almost exclusively, in the free use of purgatives and saline diuretics, and, notwithstanding the general character of the dropsy, the enormous quantity of the effused liquid, and the gravity of at least some of the symptoms, the patient, on the 20th, was evidently better, and so rapid was the convalescence, that on the 29th, only about sixteen days from the inception of his illness, every trace of swelling had disappeared; the heart-sounds were again distinct and healthy; vesicular murmur normal; cough almost entirely gone; breathing free and easy; in short, apart from some remaining debility, the recovery was perfect and complete.

In the last three reported cases can we, with due regard to the clinical history of each, charge the dropsy to renal disease? I cannot think so. Was the fault in anæmia? Certainly not, for in neither case was that condition present, even in a limited degree. Did the trouble depend upon lesion of the lungs, heart or liver? There were no evidences pointing to disease of any of those organs. Then if we are forced to exclude organic disease, whence comes the mechanical obstruction and upon what does it depend?

H. C. Wood, from whose article on acute dropsy (see *Am. Journ. of the Med. Sci.* for 1871) I have already drawn liberally, is of the opinion "that many cases of acute dropsies are due not to a mechanical impediment to the circulation, but to a peculiar condition of the cellular tissue, whereby its natural secretion or exhalation is enhanced, so that the water may be said to be actively thrown or drawn out from the vessels."

Is the theory of Dr. Wood correct; are his premises tenable? If so, I think it must be true that prior to the abnormal excitation of the cellular membrane, upon which, he claims, the effusion depends, there must have occurred certain morbid blood-changes, and that said morbid condition of the blood, whatever may be its nature, is the *direct* and primary factor in the production of the cellular irritation. If we admit the existence of acute dropsy, independent of organic trouble, does it not follow as a necessary sequence that we must admit the correctness of the idiopathic theory? And if this latter theory is accepted, upon what principle are we to account for the serous infiltration? On what particular pathological condition does the effusion depend? In the solution of this question I am free to admit that I have no theory to advance.

